# Increased and decreased cortical thickness and unaltered amygdala nuclei in patients at clinical-high risk of psychosis

**DOI:** 10.1192/j.eurpsy.2023.1157

**Published:** 2023-07-19

**Authors:** A. S. Tomyshev, A. Dudina, M. Omelchenko, E. Abdullina, I. Lebedeva, V. Kaleda

**Affiliations:** 1 Laboratory of Neuroimaging and Multimodal Analysis; 2 Department of Youth Psychiatry, Mental Health Research Center, Moscow, Russian Federation

## Abstract

**Introduction:**

There is much evidence of grey matter alterations in subjects at clinical-high risk of psychosis (CHR). Although, to the best of our knowledge, no studies have analyzed both cerebral cortex and amygdala nuclei morphometry alterations in CHR individuals.

**Objectives:**

The aim of the study was to explore cortical thickness and amygdala nuclei morphometric characteristics in CHR patients.

**Methods:**

Nineteen right-handed male patients (17-24 years, mean age 21.1 ± 2.1) fulfilling CHR criteria and 20 matched healthy controls (18-24 years, mean age 21.1 ± 1.8) underwent T1-weighted structural MRI at 3T Philips scanner. Images were processed via FreeSurfer 7.0. Cortical thickness (according to Desikan atlas) and volumes of 9 separate amygdala nuclei bilaterally were compared between groups. The morphometry data, SOPS, HDRS (Hamilton Depression Rating Scale) scores and chlorpromazine equivalents were included in correlation analysis.

**Results:**

Compared to healthy controls, patients showed decreased cortical thickness in the left [F(1, 36) = 10.8, p = 0.002; Cohen’s *d* = −1.1, 95% CI: −1.8 to −0.4] and right [F(1, 36) = 10.5, p = 0.003; Cohen’s *d* = −1.0, 95% CI: −1.7 to −0.3] postcentral gyri, and increased cortical thickness in the right posterior cingulate [F(1, 36) = 9.9, p = 0.003; Cohen’s *d* = 1.0, 95% CI: 0.3 to 1.6] and the right rostral anterior cingulate gyri [F(1, 36) = 12.2, p = 0.001; Cohen’s *d* = 1.1, 95% CI: 0.4 to 1.8]. No changes in any amygdala nuclei were detected. No correlations between altered cortical thickness, HDRS, SOPS or chlorpromazine equivalents were revealed.

**Image:**

**Figure:**
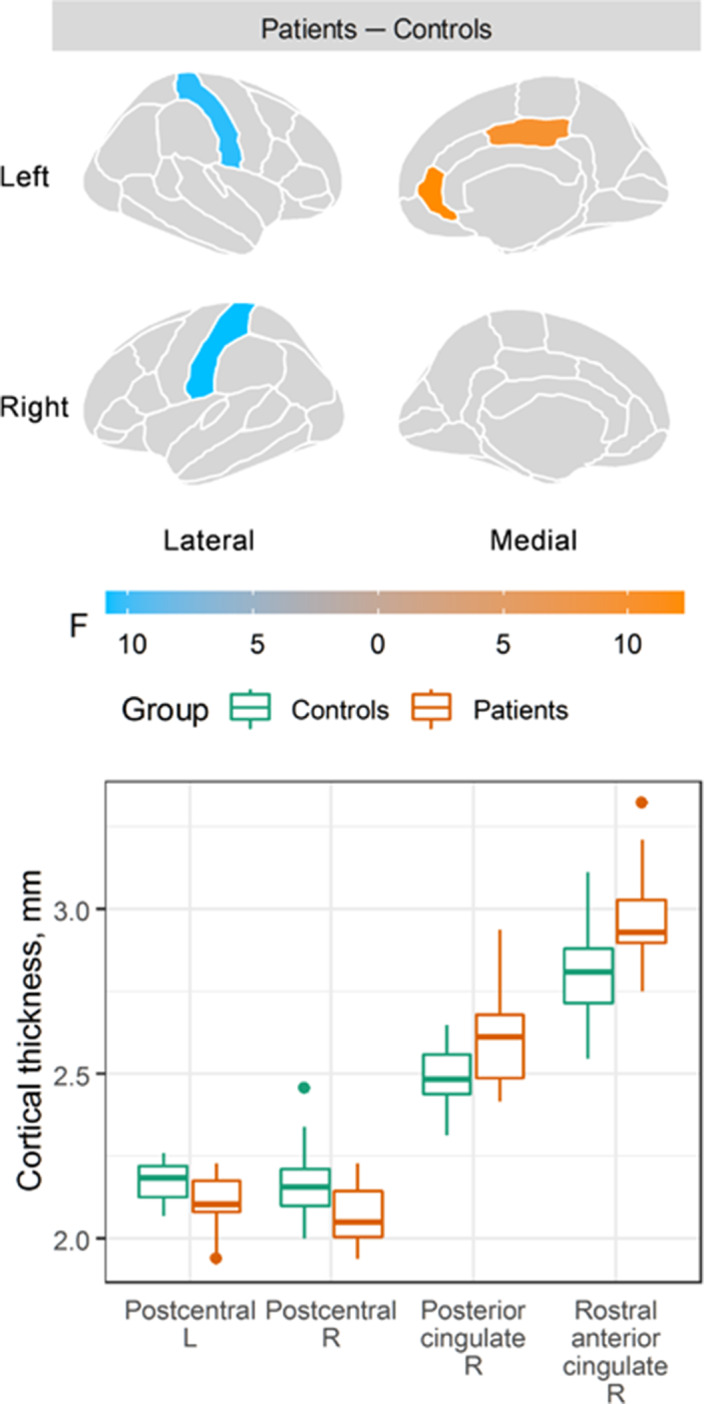

**Conclusions:**

The current findings suggest that volumetric characteristics of amygdalar complex are unaffected in the CHR state. The results have some inconsistency with our previous findings (Tomyshev *et al*. Psychiatry Res Neuroimaging. 2019; 289 26-36), which revealed only a decrease in cortical thickness in CHR individuals. However, the cross-sectional design of the current study and the lack of correlations between cortical thickness and clinical symptoms do not allow to conclude definitely whether the revealed higher cortical thickness can represents some resilience mechanisms, which will be elucidated via further research.

*This study was supported by RSFBR 22-15-00437*

**Disclosure of Interest:**

None Declared

